# A case of lymphatic flow evaluation using indocyanine green fluorescence imaging for recurrence of anastomotic site after laparoscopic right hemicolectomy

**DOI:** 10.1186/s40792-023-01741-5

**Published:** 2023-10-16

**Authors:** Keita Sato, Yosuke Yamauchi, Koji Takahashi

**Affiliations:** Department of Surgery, Ise Red Cross Hospital, 1-471-2 Funae, Ise City, Mie 516-8512 Japan

**Keywords:** Implantation, Indocyanine green (ICG), Anastomotic recurrence, ICG fluorescence imaging

## Abstract

**Background:**

Anastomotic recurrence of colorectal cancer is rare, but reoperation improves prognosis. However, there is no clear evidence regarding the extent of dissection, and there are few reports on the details of surgery. We used intraoperative lymphatic flow imaging with indocyanine green (ICG) fluorescence as a reference to determine the range of additional resection.

**Case presentation:**

The patient was a 75-year-old man who underwent laparoscopic right hemicolectomy and extracorporeal functional terminal anastomosis for ascending colon cancer 4 years ago. Histopathological examination revealed a well-differentiated tubular adenocarcinoma, T4aN0M0, pathological stageIIB. During follow-up, anemia was observed, and colonoscopy indicated anastomotic recurrence, so additional laparoscopic resection was performed. Intraoperatively, ICG was injected into the anastomotic site, and the operation proceeded under near-infrared light observation. Lymphatic vessels along the middle colonic artery were visualized down to the root of the vessel. Using this as an indicator, the vessel was ligated from the root. Using the fact that the lymphatic vessels were also depicted in the small intestinal mesentery on the oral side of the anastomosis as an indicator, the small intestine and mesentery were resected about 7 cm from the anastomosis.

**Conclusions:**

The optimal surgical approach for anastomotic recurrence of colorectal cancer has not been defined. Intraoperative ICG fluorescence imaging can provide images of lymphatic flow from the site of recurrence and may be an indicator of lymph node dissection in the case of anastomotic recurrence.

## Background

Anastomotic recurrence is relatively rare among local recurrences after colorectal cancer surgery [1, 2]. This is explained by inadequate resection margins and occult lymph node involvement, but may also be due to intraoperative transplantation of detached malignant cells. Recurrence at the stapled anastomosis site is generally caused by the transplantation of viable cancer cells into the lumen during end-to-end anastomosis (EEA) stapling [3, 4]. Although implantation, in which free tumor cells in the intestinal tract are transplanted into the submucosa, has been implicated, the appropriate range of dissection for recurrent cases has not been described. In this case, we used intraoperative imaging using ICG fluorescence to determine the extent of additional resection and dissection for re-resection of anastomotic recurrence after surgery for ascending colon cancer. We report suggestive results in oncologic evaluation for reoperation.

## Case presentation

A 75-year-old patient underwent laparoscopic right hemicolectomy for ascending colon cancer 4 years ago. The patient underwent laparoscopic resection of the ileocecal artery and the right branch of the middle colonic artery with D3 dissection. Functional end-to-end anastomosis (FEEA) was performed using automated anastomosis devices. Histopathological findings of the primary tumor were type 2, well-differentiated ductal adenocarcinoma, pSE, (pT4a), INFβ, ly1, v2, pN0, pH0, pP0, pM0, and pStage IIB, *and his cancer defined as RAS wild tumor*. The distance to the tumor at the resection margin was pathological proximal margin (PM0) at the proximal dissected edge of 90 mm, pDM0 (distal margin) at the distal dissected edge of 133 mm, and no cancer invasion on the lateral dissected surface, indicating a curative grade A.

After that, he was referred to our hospital because of a neoplastic lesion near the anastomosis between the ileum and the transverse colon detected by computed tomography colonography.

**Fig. 1:**
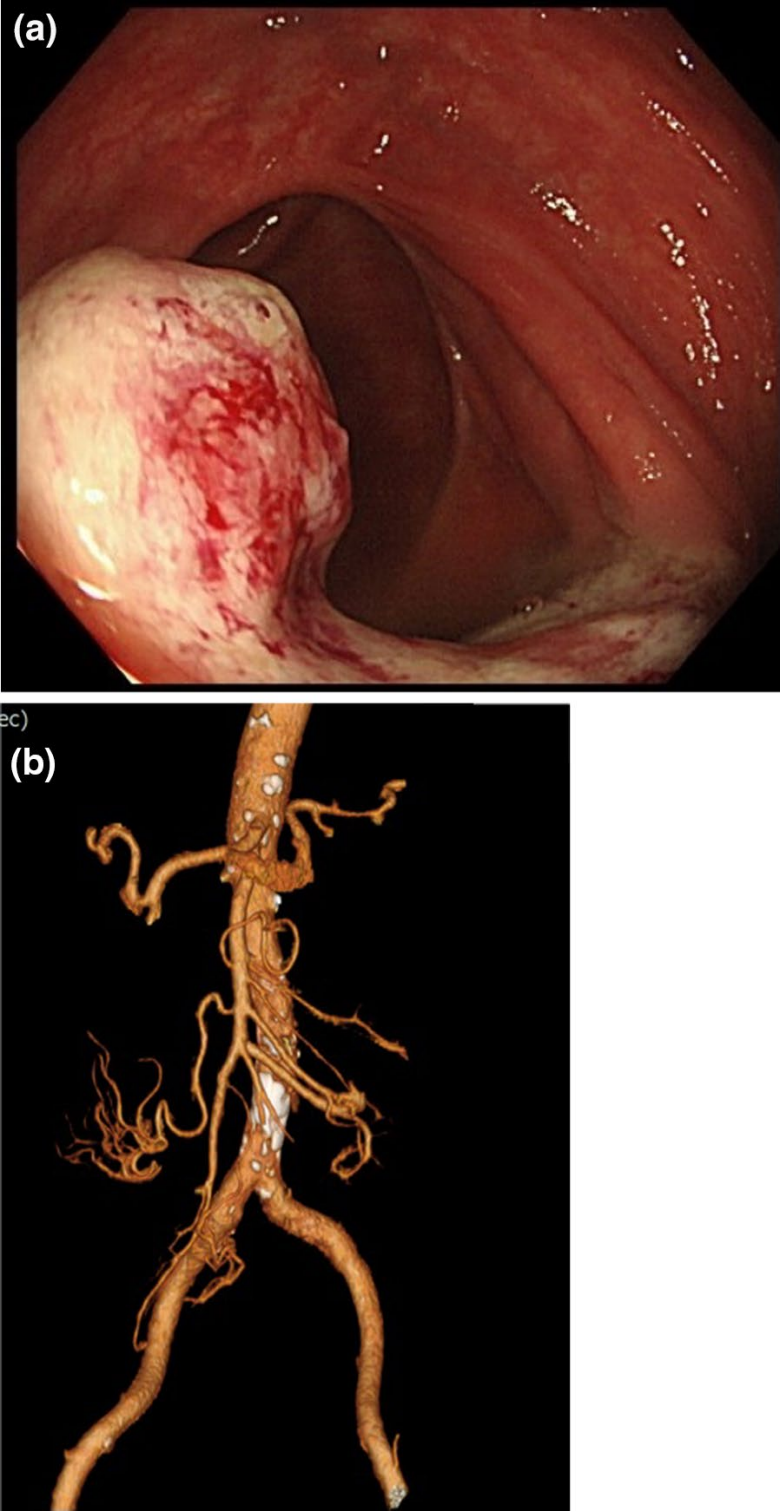
**a** Colonoscopy findings. A mossy-white, elevated lesion was found at the anastomosis of the ileum-transverse colon. **b** Preoperative 3D-CT angiography showed that the root of the middle colonic artery was preserved, which is responsible for blood flow on the anal side of the anastomosis

Colonoscopy (Fig. 1a) revealed an elevated lesion covered with moss white on concentric circles at the anastomosis of the ileum and transverse colon. Preoperative 3D-CT angiography showed that the root of the middle colonic artery was preserved, which is responsible for blood flow on the anal side of the anastomosis (Fig. 1b). Biopsy of the same area revealed growth of highly differentiated tubular adenocarcinoma.

**Fig. 2:**
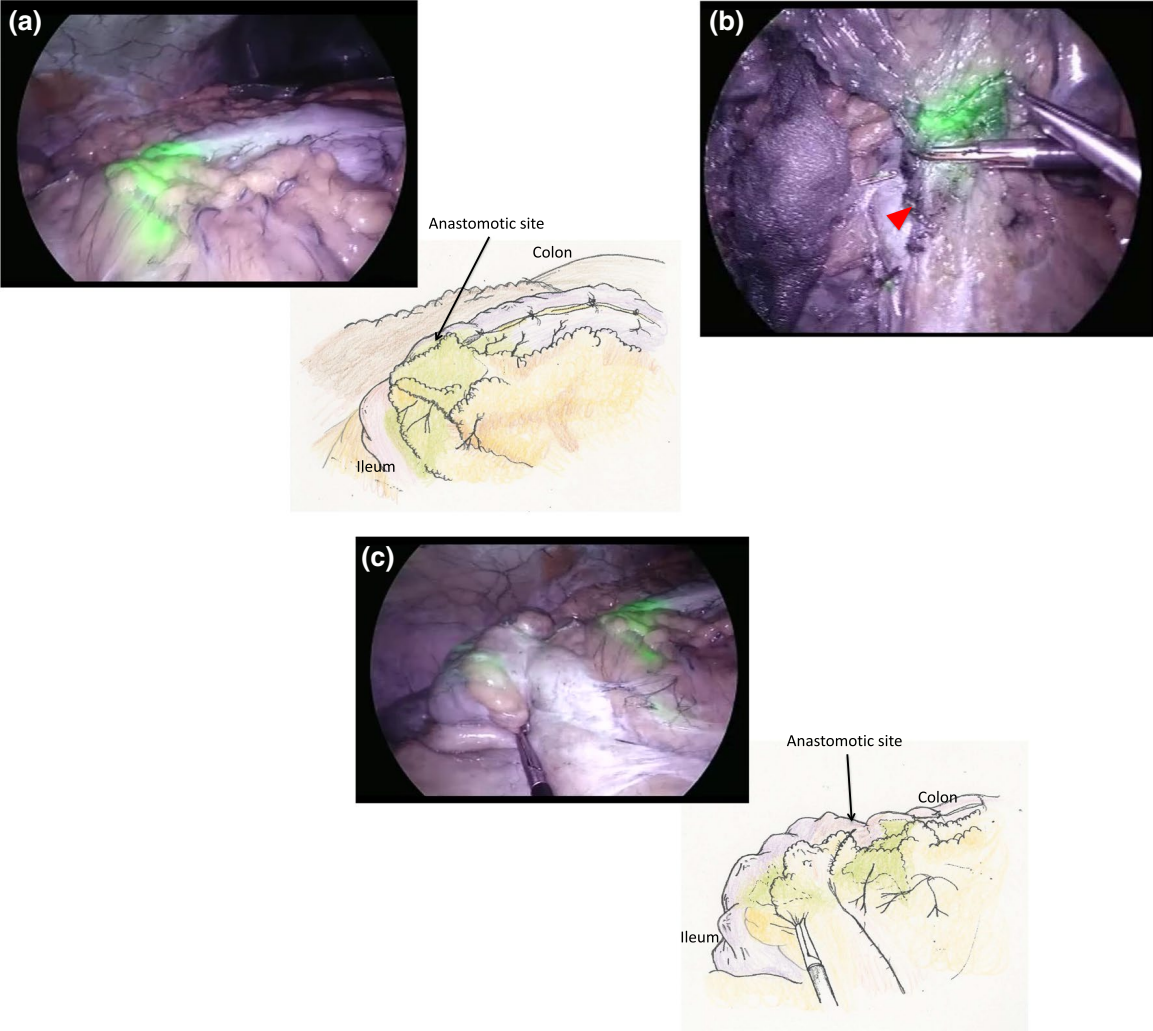
Fluorescence imaging after ICG injection. **a** Lymphatic flow from the anastomosis into the surrounding mesentery was identified under ICG fluorescence imaging. **b** The lymph node delineated by ICG fluorescence method at the root of the middle colonic artery (arrow) was dissected. **c** Detailed observation with forceps revealed that the ICG had entered the mesentery of the small intestine on the oral side of the anastomosis. The mesentery of the small intestine is greenish in color

The patient was diagnosed with anastomotic recurrence 4 years postoperatively and underwent additional laparoscopic resection. ICG was diluted to 5 mg/ml and administered at the beginning of the procedure with an endoscopic 25-gauge needle, 1 ml of ICG was administered from the serosal surface to the submucosa on the anal side about 10 mm of the mass of the anastomotic site, and the operation proceeded with near-infrared light observation (Fig. 2a). Lymphatic vessels along the middle colonic artery were delineated to the root of the vessel. Using this as an indicator, the vessel was ligated at its base (Fig. 2b). In addition, since the ICG was found to have entered the mesentery of the small intestine on the oral side of the anastomosis (Fig. 2c), the small intestine and mesentery were resected about 7 cm from the anastomosis. Anastomoses were performed extracorporeally, wiped thoroughly with povidone-iodine, and then anastomosed with hand sawn.

**Fig. 3:**
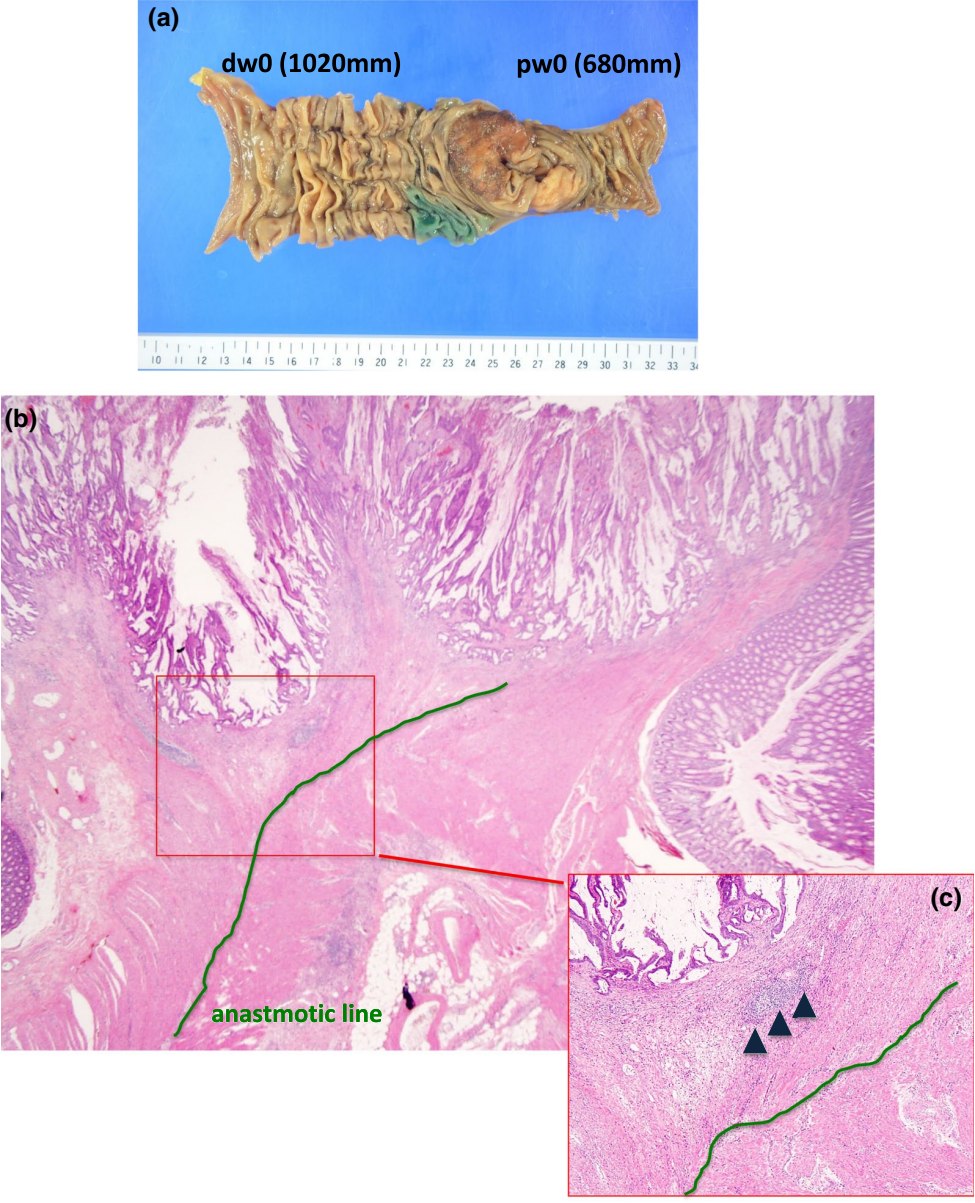
Gross and microscopic findings of the resected specimen. **a** Macroscopically, a 3.8 × 3.6 cm raised lesion was observed at the anastomosis. **b**, **c** Microscopic examination revealed an anastomotic area where the continuity of the intestinal muscular layer was interrupted (**b** solid line). Tumor cell growth was observed on the anorectal side of the anastomosis (arrowhead, **c**)

Macroscopically, a 3.8 × 3.6 cm raised lesion was observed at the anastomosis (Fig. 3a). No lymph node metastasis was observed. The histological type was complex ductal adenocarcinoma with depth SS, ly1a, v0, N0, pn0, pw0 (680 mm), dw0 (1020 mm). Microscopic examination revealed an anastomosis where the continuity of the muscular layer was interrupted (Fig. 3b), and tumor cell growth was observed on the anal side from this point (arrowhead, Fig. 3c), which confirmed the diagnosis of anastomotic recurrence.

## Discussion

In the sites of recurrence after curative resection of all colorectal cancers, intestinal anastomosis is relatively rare, accounting for about 1% [5]. According to past case reports of anastomotic recurrence, 9.8% of the anastomoses in the initial surgery were hand-sewn, whereas 90.2% were using a stapler [1]. For rectal cancer, the distal margin on the anal side is secured over a shorter distance than for colon cancer, but there is no difference in the recurrence rate based on the location of the primary tumor [1, 6, 7]. On the other hand, cases with a depth of T3 or greater, positive lymph nodes, or advanced cancer with a tumor diameter of 50 mm or greater or an annular circumference of 80% or greater have been suggested to be at risk for anastomotic recurrence [7, 8].

The possibility of pushing intestinal floating cancer cells into the submucosa during needle insertion [9] has long been pointed out for intestinal anastomosis in colorectal cancer surgery, whether by instrumented or hand-sewn anastomosis. However, the anastomosis with an automatic anastomosis is characterized by a large number of staples, a long staple line [7], and residual metal in the intestinal wall at the anastomosis site, but it is unclear whether this directly increases the risk of tumor cell transplantation.

Although there is no established guideline for additional resection at the time of anastomotic recurrence [5, 10], it is necessary to include lymphatic flow of the recurrent tumor in the range of dissection and not to cause another implantation. In addition, when anastomosis is performed with a stapler, the B-shaped formation of the staple extends to all layers of the intestinal wall, which may cause intestinal free cancer cells to implant in the submucosa, muscle layer, or even deeper layers. Thus, there is a reasonable possibility that recurrent tumors may arise from these layers. This raises the clinical question of the extent to which recurrent tumor should be considered as a tumor invasion, i.e., whether vertical lymph node dissection should be performed, when re-resection is performed.

Regarding horizontal dissection, previous reports indicate that the frequency of cancer cells detected in the intestinal mucosa prior to anastomosis for colorectal cancer was 12.5% for oral and 21.2% for anal incision [8, 11, 12]. It has also been reported that there is a high density of free cancer cells in the intestinal tract within 5 cm of the tumor [13]. The fact that it has been noted that free cancer cells are also found on the oral side of the anastomosis [11, 12] may be an important factor in determining the extent of horizontal dissection, including the oral side, in surgery at the time of recurrence.

There are many case reports of reoperation for colorectal cancer that recurred at the anastomosis after initial FEEA [14–17]. However, descriptions of reoperation techniques are limited to “partial resection” and the like, and there are few detailed descriptions of the extent of lymphatic dissection. In particular, none of the case reports mentioned the extent of vertical dissection. In our case, central vascular ligation and D3 dissection, including 7 cm of mesentery on the oral side of the small intestine, were performed based on information from ICG fluorescence methods. In situations where the appropriate extent of dissection is unknown, it is meaningful to visualize lymphatic flow from the anastomosis as an indicator of dissection. Our case is the first report of the use of ICG in surgery for anastomotic recurrence.

On the other hand, current intraoperative use of ICG is limited, and is generally intended primarily to assess perfusion of the colonic facet to reduce the risk of anastomotic leakage. Besides, it is also used as a valuable tool to clarify lymphatic drainage when performing iliac lymph node chain and lateral lymph node dissection [18].

Although this method has not been shown to be useful in determining the extent of lymph node dissection, we believe it can help clarify drainage of lymphatic flow from the anastomosis and help determine the extent of dissection during additional resection of anastomotic recurrences.

## Conclusion

In reviewing past reports of colon anastomotic recurrences, many considered a causal relationship to the initial surgery, but none mentioned the appropriate range of additional resection. Intraoperative imaging with ICG fluorescence may help to evaluate lymphatic flow in the vicinity of the tumor and to determine the appropriate horizontal and vertical dissection area for additional resection.

## Data Availability

Not applicable.

## Abbreviations

ICG: Indocyanine green
CT: Computed tomography
FEEA: Functional end-to-end anastomosis
PM: Proximal margin
DM: Distal margin
